# Clinical, radiological and histopathological features of patients with familial pulmonary fibrosis

**DOI:** 10.1186/s12931-024-02864-5

**Published:** 2024-06-12

**Authors:** Hanna Jaula, Lauri Mattila, Elisa Lappi-Blanco, Johanna Salonen, Hannu Vähänikkilä, Lauri Ahvenjärvi, Jukka S. Moilanen, Outi Kuismin, Terttu Harju, Riitta Kaarteenaho

**Affiliations:** 1 Research Unit of Biomedicine and Internal Medicine, University of Oulu, Oulu, Finland; 2 Center of Internal Medicine and Respiratory Medicine, and Medical Research Center Oulu, Oulu University Hospital, Oulu, Finland; 3 Department of Diagnostic Radiology, Oulu University Hospital, Oulu, Finland; 4 Department of Pathology, Oulu University Hospital, Oulu, Finland; 5 Department of Pathology, Research Unit of Translational Medicine, University of Oulu, Oulu, Finland; 6 Northern Finland Birth Cohorts, Arctic Biobank, Infrastructure for Population studies, Faculty of Medicine, University of Oulu, Oulu, Finland; 7 Department of Clinical Genetics and Medical Research Center Oulu, Oulu University Hospital, Oulu, Finland; 8 Research Unit of Clinical Medicine, University of Oulu, Oulu, Finland

**Keywords:** Familial pulmonary fibrosis, Interstitial lung disease, Comorbidity, Histopathology, Radiology, Survival, Risk prediction model

## Abstract

**Background:**

In familial pulmonary fibrosis (FPF) at least two biological relatives are affected. Patients with FPF have diverse clinical features.

**Research question:**

We aimed to characterize demographic and clinical features, re-evaluate high-resolution computed tomography (HRCT) scans and histopathology of surgical lung biopsies, assess survival and investigate the suitability of risk prediction models for FPF patients.

**Study design:**

A retrospective cohort study.

**Methods:**

FPF data (*n* = 68) were collected from the medical records of Oulu University Hospital (OUH) and Oulaskangas District Hospital between 1 Jan 2000 and 11 Jan 2023. The inclusion criterion was pulmonary fibrosis (PF) (ICD 10-code J84.X) and at least one self-reported relative with PF. Clinical information was gathered from hospital medical records. HRCT scans and histology were re-evaluated.

**Results:**

Thirty-seven (54.4%) of the patients were men, and 31 (45.6%) were women. The mean ages of the women and men were 68.6 and 61.7 years, respectively (*p* = 0.003). Thirty-seven (54.4%) patients were nonsmokers. The most common radiological patterns were usual interstitial pneumonia (UIP) (51/75.0%), unclassifiable (8/11.8%) and nonspecific interstitial pneumonia (NSIP) (3/4.4%). Pleuroparenchymal fibroelastosis (PPFE) was observed as a single or combined pattern in 13.2% of the patients. According to the 2022 guidelines for idiopathic pulmonary fibrosis (IPF), the patients were categorized as UIP (31/45.6%), probable UIP (20/29.4%), indeterminate for UIP (7/10.3%) or alternative diagnosis (10/14.7%). The histopathological patterns were UIP (7/41.2%), probable UIP (1/5.9%), indeterminate for UIP (8/47.2%) and alternative diagnosis (1/5.9%). Rare genetic variants were found in 9 patients; these included telomerase reverse transcriptase (*TERT*, *n* = 6), telomerase RNA component (*TERC*, *n* = 2) and regulator of telomere elongation helicase 1 (*RTEL1*, *n* = 1). Half of the patients died (*n* = 29) or underwent lung transplantation (*n* = 5), with a median survival of 39.9 months. The risk prediction models composite physiology index (CPI), hazard ratio (HR) 1.07 (95.0% CI 1.04–1.10), and gender-age-physiology index (GAP) stage I predicted survival statistically significantly (*p*<0.001) compared to combined stages II and III.

**Conclusions:**

This study confirmed the results of earlier studies showing that FPF patients’ radiological and histopathological patterns are diverse. Moreover, radiological and histological features revealed unusual patterns and their combinations.

## Background

Pulmonary fibrosis (PF) is a devastating interstitial lung disease (ILD) that causes lung parenchymal scarring. At least two biological relatives are affected in familial pulmonary fibrosis (FPF) [1, 2]. FPF is a strong risk factor for idiopathic pulmonary fibrosis (IPF), but FPF has also revealed various other forms of ILD [3, 4]. Compared with IPF patients without a familial burden, FPF patients are often younger and have worse survival [4, 5].

There is a wide range of reported familial pulmonary fibrosis cases in the studies, ranging from 3.7% to 35.9%, depending on the inclusion criteria, as discussed in a recent editorial by Dickinson and Lucas [6]. Twenty-five percent of FPF and five percent of sporadic IPF cases have causative genetic variants, of which the most common are in telomere-related genes causing telomere shortening [7]. The most common telomere-related variants are in telomerase reverse transcriptase (*TERT)*, regulator of telomere elongation helicase 1 (*RTEL1),* poly(A)-specific ribonuclease *(PARN)* and telomerase RNA component (*TERC)* [7, 8]. Clinical diagnoses and radiological patterns have been shown to be diverse since almost every second patient has IPF, but there are also other forms of ILD, such as unclassifiable PF, pleuroparenchymal fibroelastosis (PPFE) and chronic hypersensitivity pneumonitis (CHP) [9]. Furthermore, ILD patients with shortened telomeres have radiologically and histologically atypical findings, which results in diagnostic challenges and thus emphasizes the need for multidisciplinary team meetings [10].

We found only few studies about disease course prediction models in telomerase diseases or FPF. Planas-Cerezales et al. investigated the suitability of survival prediction model (Gender-Age-Physiology, GAP) in IPF patients and found poorer survival in shortened telomere group under 60 years old although they were classified to group GAP I [11]. Manali et al. investigated GAP within a cohort of suspected hereditary PF which consisted of 63.3% of FPF patients showing association between increased risk of death and higher GAP index and stage [12]. However, there are no previously published studies on GAP or the composite physiology index (CPI) in FPF patients alone.

Arterial hypertension (HT), coronary artery disease (CAD), diabetes, hypercholesterolemia (HC), emphysema, gastroesophageal reflux disease (GERD) and obstructive sleep apnea (OSA) have been the most common comorbidities reported in IPF, unclassifiable ILD and CHP patients [13–18]. Bennet et al. revealed that the most common comorbidities were arterial hypertension, osteoporosis, hypercholesterolemia and GERD in a cohort of 47 FPF patients. Thyroid diseases were also found to be common, especially in patients inconsistent with usual interstitial pneumonia (UIP) patterns [19]. Krauss et al. observed pulmonary hypertension in 26% of patients and OSA and pulmonary embolism in 7% of the patients in a study that included 27 FPF patients [5].

FPF individuals in Finland were studied more than two decades ago by Hodgson et al. when the prevalence was calculated to be 5.9 per million people [20]. In the above-mentioned study, the clinical findings between familial and sporadic cases seemed to be similar, although FPF patients were slightly younger without male predominance [20]. Our aims were to characterize the clinical, radiological and histological features of FPF in a Northern Finnish cohort and moreover, to re-evaluate high-resolution computed tomography (HRCT) scans and surgical lung biopsies according to the 2022 International IPF Guidelines [21, 22]. We also studied the suitability of survival prediction models for FPF patients.

## Study design and methods

### Patients and data selection

The retrospective cohort study population of 68 patients with FPF was collected from the electronic medical records of Oulu University Hospital (OUH) and Oulaskangas District Hospital dating back approximately 25 years using the International Classification of Diseases 10th edition (ICD-10) code J84.X and the patients listed in the ILD multidisciplinary group meetings between 1 Jan 2000 and 11 Jan 2023. The overall number of patients coded by J84.X in both hospitals from 2000 to 2021 was 1.039 (unpublished data).

The inclusion criterion was a patient with PF who had at least one self-reported relative with PF. Patients with sarcoidosis were not included. Clinical information was gathered using a specifically designed form and included age at the first visit, gender, smoking habits, occupation, exposure, presence of inspiratory crackles or finger clubbing at the time of diagnosis, body mass index (BMI), genetic testing if available, number of PF patients in the family and whether the relative was a first, second or third degree of relative. Pulmonary symptoms and comorbidities, including CAD, HT, HC, GERD, connective tissue disease (CTD), asthma, chronic obstructive pulmonary disease (COPD), pulmonary embolism (PE), lung cancer and other malignancies, diabetes, heart failure (HF) for any reason, cerebral infarction (CI), OSA, liver dysfunction, arteriosclerosis (ASO), thyroid diseases and atrial fibrillation, were recorded. The results of pulmonary function tests (PFTs), i.e., forced vital capacity (FVC), forced expiratory volume in one second (FEV1) and diffusing capacity for carbon monoxide (DLCO), were collected at the time of diagnosis. At least three months of anemia, macrocytosis, leucopenia or thrombocytopenia were observed if no obvious cause was noticed. Histological reports of samples from surgical lung biopsies and autopsies were reviewed. Surgical lung biopsies were performed between 2006 and 2019 according to the recommendations of that time. Clinical diagnoses were gathered as they were established in clinical practice and in a multidisciplinary team meeting. Information on medication for PF was also gathered. Patients who had used antifibrotics for at least three months (i.e., nintedanib or pirfenidone) were considered antifibrotic drug users. Patients with a smoking history of less than three pack-years were classified as nonsmokers. Survival time was calculated from the first visit to the pulmonary clinic as the baseline, and the endpoint was death or lung transplantation. The overall survival time was calculated from the first visit to the pulmonology clinic due to PF to the date of death, lung transplantation or last follow-up (11 Jan 2023).

### Radiological re-evaluation

The HRCT scans were re-evaluated by experienced thoracic radiologists (LM, LA) and chosen as the one closest to the baseline when available. UIP patterns were re-evaluated according to the 2022 IPF guidelines, and ILD was classified according to the 2013 guidelines of idiopathic interstitial pneumonia (IIP) [22, 23]. The radiological features were categorized by IPF guidelines as UIP, probable UIP, indeterminate for UIP and alternative diagnosis [21–23]. For statistical analyses, the patients were divided into two study groups (i.e., UIP and non-UIP). The UIP group included both UIP and probable UIP patterns, and the non-UIP group included indeterminate UIP patterns and other pulmonary fibroses, such as unclassifiable PF, nonspecific interstitial pneumonia (NSIP), organizing pneumonia (OP), hypersensitivity pneumonia (HP), PPFE and respiratory bronchiolitis interstitial lung disease (RB-ILD). Agreement between the radiologists’ re-evaluations was expressed as a kappa value (κ).

### Histopathological re-evaluation

Surgical lung biopsies and autopsies were re-evaluated by an experienced pathologist (EL-B). Biopsies were categorized as UIP, probable UIP, indeterminate for UIP or alternative diagnosis according to the 2022 IPF guidelines [21, 22]. In addition, a detailed evaluation of additional histological features was performed.

### Risk prediction models

Gender-Age-Physiology index was calculated by using gender and baseline age, FVC% and DLCO% [24]. Patients were categorized into three stages according to the total score: GAP I (total score 0‒3), II (total score 4‒5) and III (total score 6‒8). The composite physiology index (CPI) was calculated by the formula 91 – (0.65 × DLCO%) – (0.53 × FVC%) + (0.34 × FEV1%) [25].

### Statistical analyses

Categorial variables are shown as frequencies, n (%), and Pearson chi-square or Fisher’s Exact Test were used for testing differences between variables. The Shapiro‒Wilk test was used to test the normality of the data, and the independent samples t test was used to compare continuous variables. A Kaplan-Meier curve was drawn to estimate median survival time, and the log rank test was used to calculate the difference between median survival times. Survival time is expressed as months. Cox proportional hazards were used to obtain hazard ratios (HRs) and 95% confidence intervals (95% CIs) to investigate the associations between survival and these variables. *P* values < 0.05 were considered to indicate statistical significance. Agreement between the radiologists’ re-evaluations was expressed as a kappa value (κ). Values of κ 0.41–0.60 were considered moderate, and κ values 0.61–0.80 were considered good agreement [26]. All the data were analyzed by IBM Statistics SPSS software version 29.0, and graphics were created by OriginPro, Version 2022b. OriginLab Corporation, Northampton, MA, USA.

## Results

### Patient characteristics

Sixty-eight patients with mean age of 64.4 years were included in the study. Patient characteristics are shown in Table 1. Thirty-seven (54.4%) patients were nonsmokers, 25 (36.8%) were ex-smokers, and 6 (8.8%) were current smokers. Shortness of breath during exercise was the most common symptom at baseline in 42 (61.8%) patients. Other common symptoms were cough in 36 (52.9%) patients and mucus production in 23 (33.8%) patients. At the time of diagnosis, ten patients (14.7%) were asymptomatic. Inspiratory crackles were heard by stethoscope auscultation in 57 (83.8%) patients. Seventeen (25%) patients had finger clubbing.

**Table 1:** Characteristics and clinical diagnoses of the patients with familial pulmonary fibrosis

**Characteristics**	***N*=68 (%)**
Age (y)	64.4 ± 9.6
Nonsmoker	37 (54.4)
Pack-years	9 ± 11.9
Pulmonary function test results	
FVC (%)^a^	79.4 ± 19.5
FEV1 (%)^a^	86.2 ± 20.1
FEV1/FVC^b^	84.9 ± 5.8
DLCO (%)^c^	61.3 ± 18.4
BMI	27.9 ± 5.59
Clinical diagnosis	
IPF	46 (67.6)
Unclassifiable	9 (13.2)
CTD-ILD	7 (10.3)
PPFE	2 (2.9)
NSIP	2 (2.9)
COP	1 (1.5)
CHP	1 (1.5)

*BMI* body mass index, *CHP* chronic hypersensitivity pneumonitis, *COP* cryptogenic organizing pneumonia, *CTD-ILD* connective tissue disease associated interstitial lung disease, *DLCO* diffusion capacity to carbon monoxide, *FEV1* forced expiratory volume in one second, *FVC* forced vital capacity, *IPF* idiopathic pulmonary fibrosis, *NSIP* nonspecific interstitial pneumonia, *PPFE* pleuroparenchymal fibroelastosis

^a^Missing two cases

^b^Missing seven cases

^c^Missing three cases

Fifty (73.5%) patients were discussed in multidisciplinary meetings. The most common clinical diagnose was IPF (*n* = 46/67.6%) (Table 1).

Twenty-seven (39.7%) of the patients were receiving antifibrotic medication and 27 (39.7%) patients did not have medication for PF. Seven (10.3%) patients had prednisolone treatment and two patients had azathioprine in addition to prednisolone. Five patients (7.4%) were treated with prednisolone, azathioprine and N-acetylcysteine.

### Gender

Characteristics of women and men are presented in Table 2. Thirty-one (83.8%) of the men had UIP compared to 20 (64.5%) of the women, *p* = 0.068. Furthermore, 27 (73%) of the men had IPF and 19 (61.3%) of the women, *p* = 0.305.

**Table 2:** Characteristics of the familial pulmonary fibrosis patients by the gender

**Characteristics**	**MEN** ***N*=37 (%)**	**WOMEN** ***N*=31 (%)**	***P*-value**
**Age (y)**	61.7 ± 9.7	68.6 ± 8.1	0.003
**Nonsmoker**	16 (43.2)	21 (67.7)	0.043
**Pack-years**	12.2 ± 12.7	5.1 ± 9.7	0.013
**Pulmonary function test results**			
FVC (%)*	74.3 ± 18.0	85.1 ± 19.9	0.024
FEV1 (%)*	80.4 ± 18.3	92.7 ± 20.3	0.011
DLCO (%)**	61.4 ± 19.3	61.1 ± 17.7	NS
** FEV1/FVC**	84.9 ± 5.5	84.9 ± 6.2	NS
**BMI**	28.7 ± 4.7	26.9 ± 6.4	NS
**Death or lung transplantation**	17 (45.9)	17 (54.8)	NS
**Age at death or lung transplantation**	65.4 ± 11.1	74.0 ± 9.7	0.022

*BMI* body mass index, *DLCO* diffusion capacity to carbon monoxide, *FEV1* forced expiratory volume in one second, *FVC* forced vital capacity, *NS* not significant

^*^Missing two in men group

^**^Missing two in men group and one in women group

### Radiological re-evaluation

According to the IIP 2013 classification, the patients were categorized as UIP (51/75.0%), unclassifiable (8/11.8%), NSIP (3/4.4%), PPFE (2/2.9%), HP (2/2.9%), OP (1/1.5%) and RB-ILD (1/1.5%). Nine patients (13.2%) had a PPFE pattern, in which PPFE was combined with UIP in five patients, with NSIP in one patient and with probable UIP in one patient. In two patients, PPFE occurred as a single pattern.

**Fig. 1:**
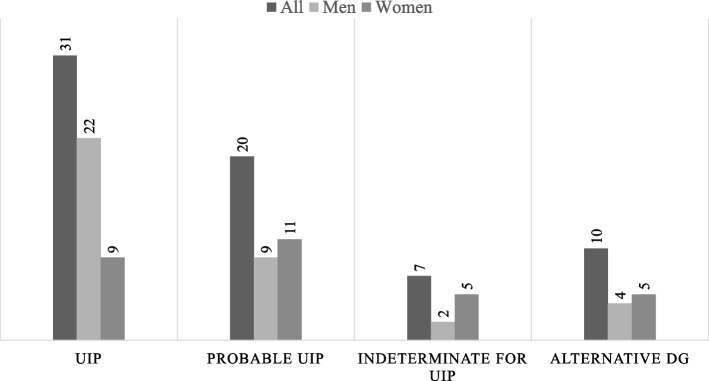
Radiologic UIP patterns according to the 2022 idiopathic pulmonary fibrosis guidelines. UIP = usual interstitial pneumonia

According to the IPF 2022 guidelines, the patients were reclassified as UIP (*n* = 31/45.6%), probable UIP (*n* = 20/29.4%), indeterminate for UIP (*n* = 7/10.3%) or alternative diagnosis (*n* = 10/14.7%) (Fig. 1). When patients were classified according to IPF guidelines and further divided into UIP and non-UIP groups, the agreement between the radiologists was moderate (κ = 0.474 and κ = 0.519, respectively). There was no significant difference in smoking between the UIP and non-UIP groups, although in the non-UIP group, the DLCO% predicted was greater (*p* = 0.035).

### Genetic findings

In 59 (86.8%) patients, a first-degree relative had ILD; in three (4.4%) patients, a second-degree relative had ILD; and in six (8.8%) patients, a third-degree relative had ILD. Twenty-three (33.8%) patients had two or more affected relatives. Twenty-four patients underwent genetic testing (BluePrint Genetics) at the Department of Clinical Genetics of Oulu University Hospital, in which six *TERT*, two *TERC* and one *RTEL1* pathogenic variant were found. In fifteen patients no known pathogenic gene variants were identified. Both patients with the *TERC* gene variant had dyskeratosis congenita, although the gene variant was heterozygous. Three patients with pathogenic gene variants had anemia, one with myelodysplastic syndrome (MDS) and another with macrocytosis. Two patients had thrombocytopenia, and four patients did not have any hematological disorders (Table 3). Patients with a pathogenic gene variant were younger than those who were tested negative (58.7 years and 64.9 years, respectively, *p* = 0.006). No other differences were found among the groups in gender, smoking status, radiological patterns, clinical diagnoses or pulmonary function tests.

**Table 3:** Cases with pathogenic gene variants

**Gene**	**Variant**	**Telomere length**	**Hematological abnormalities**	**Radiologic pattern**	**Clinical diagnosis**
*TERT*	c.2320C>T, p.Arg(774*)	Short	Thrombocytopenia	UIP, PPFE	IPF
*TERT*	c.2051A>G, p.(Asp684Gly)	Short	None	UIP	CTD-ILD
*TERT*	c.2051A>G, p.(Asp684Gly)	Short	None	UIP, PPFE	IPF
*TERT*	c.2051A>G, p.(Asp684Gly)	LL	Anemia, MDS	UIP	IPF
*TERT*	c.2051A>G, p.(Asp684Gly)	LL	None	PPFE	PPFE
*TERT*	c.2051T>C, p.(Asp684Gly)	Short	Anemia, macrocytosis	UIP	IPF
*TERC*	r.287C>G	Short	Anemia	UIP	IPF
*TERC*	r.287C>G	Short	None	UIP	IPF
*RTEL1*	c.3028C>T, p.(Arg1010*) TNFRSF13B, c.310T>C	Short	Thrombocytopenia	UIP	IPF

All the gene variants were heterozygous. *LL* lower limit of normal, *CTD-ILD* connective tissue disease associated interstitial lung disease, *IPF* idiopathic pulmonary fibrosis, *MDS* myelodysplastic syndrome, *PPFE* pleuroparenchymal fibroelastosis, *RTEL1* regulator of telomere elongation helicase 1, *TERC* telomerase RNA component, *TERT* = telomerase reverse transcriptase, *UIP* usual interstitial pneumonia

### Comorbidities

**Fig. 2:**
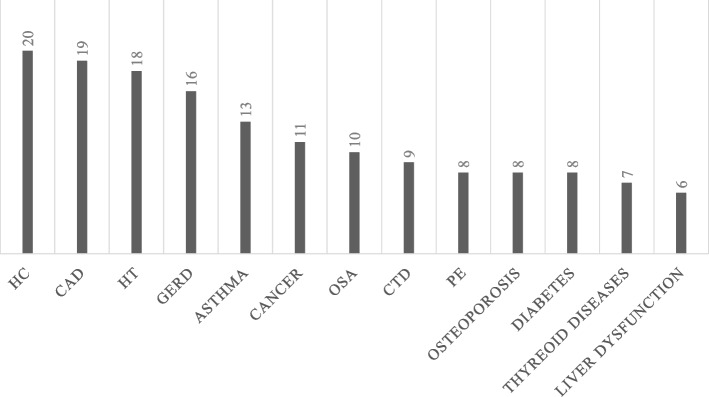
The most common comorbidities. HC = Hypercholesterolemia, CAD = Coronary artery disease, HT = Hypertension, Diabetes (type I and II), GERD = gastroesophageal reflux disease, CTD = Connective Tissue Disease, OSA = Obstructive Sleep Apnea, PE = pulmonary embolism

The most common comorbidities are shown in Fig. 2. Eleven (16.2%) patients had malignant disease, but only one patient was suspected of having malignant lung disease, although lung or pleural cancer was not histologically confirmed. Asthma was the most common obstructive airway disease in 13 (19.1%) patients, while none of them suffered from COPD. In total, nine patients (13.2%) had CTD, including eight patients with rheumatoid arthritis (RA) and one with polymyalgia rheumatica. More women (*n* = 7) had osteoporosis than men (*n* = 1, *p* = 0.019), while only men had liver dysfunction (*n* = 6, *p* = 0.028). Of the patients with liver dysfunction, one had a pathogenic gene variant (*TERT*), one was tested but no pathogenic variants were found and four were not tested. Besides osteoporosis and liver dysfunction, there were no significant differences between genders in other comorbidities (Fig. 2).

### Histopathological re-evaluation

**Fig. 3:**
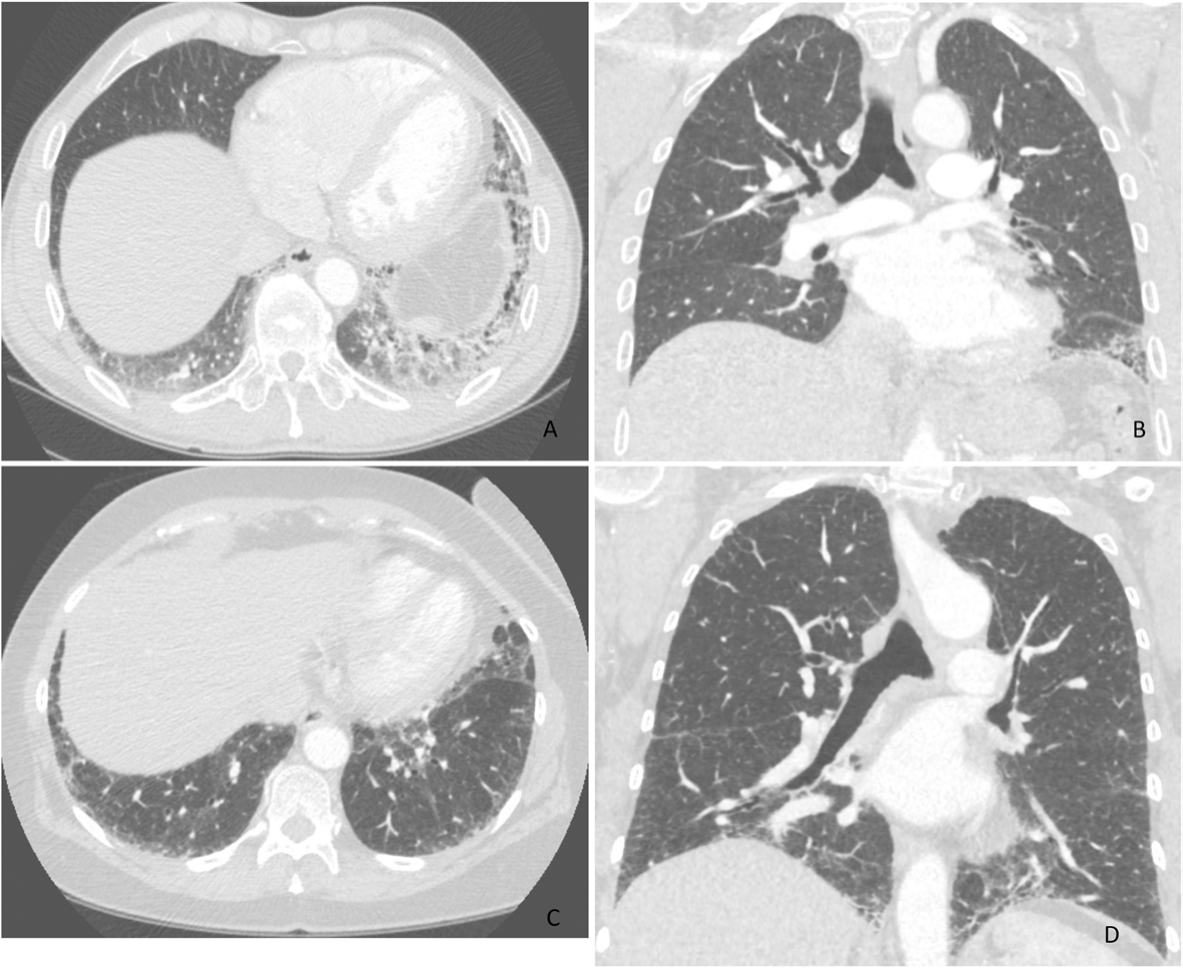
High resolution computed tomography (HRCT) scans of two patients with the *TERC* variant. **A**-**B**: HRCT scan of patient number 1 shows a left lung predominant usual interstitial pneumonia pattern with basal and peripheral distribution, reticular abnormalities, traction bronchiectasis and honeycombing. **C**-**D**: HRCT scan of patient number 2 reveals fine reticular abnormalities and traction bronchiectasis without honeycombing with basal and peripheral distribution

**Fig. 4:**
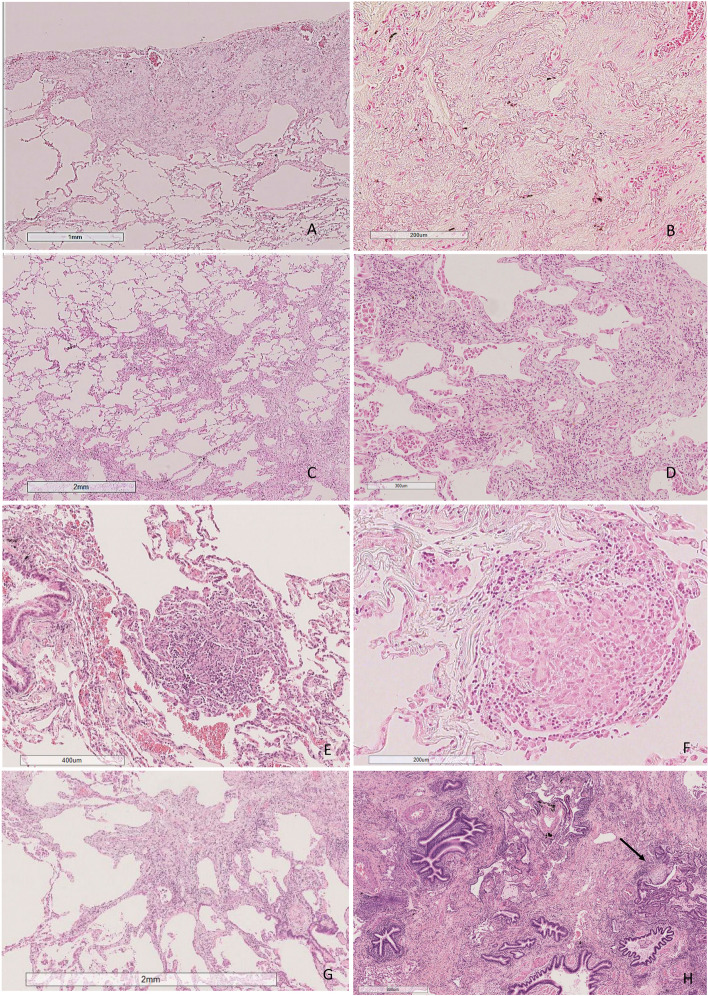
Histological images from a patient with a *TERC* variant (no 1). In the upper left lobe, there were separate subpleural or paraseptal areas similar to those of pleuroparenchymal fibroelastosis (**A**) with thick elastic fibers (**B**). There were also a few centrilobular areas with slightly fibrotic alveolar walls (**C**) and focal chronic inflammation (**D**), as well as peribronchiolar aggregations of fibromyxoid connective tissue and macrophages (**E**) and poorly formed granulomas (**F**). In the lower left lobe, fibrosis was more prominent, with centrilobular bridging fibrosis (**G**) and architectural remodeling (**H**) with occasional fibroblast foci (arrow)

**Fig. 5:**
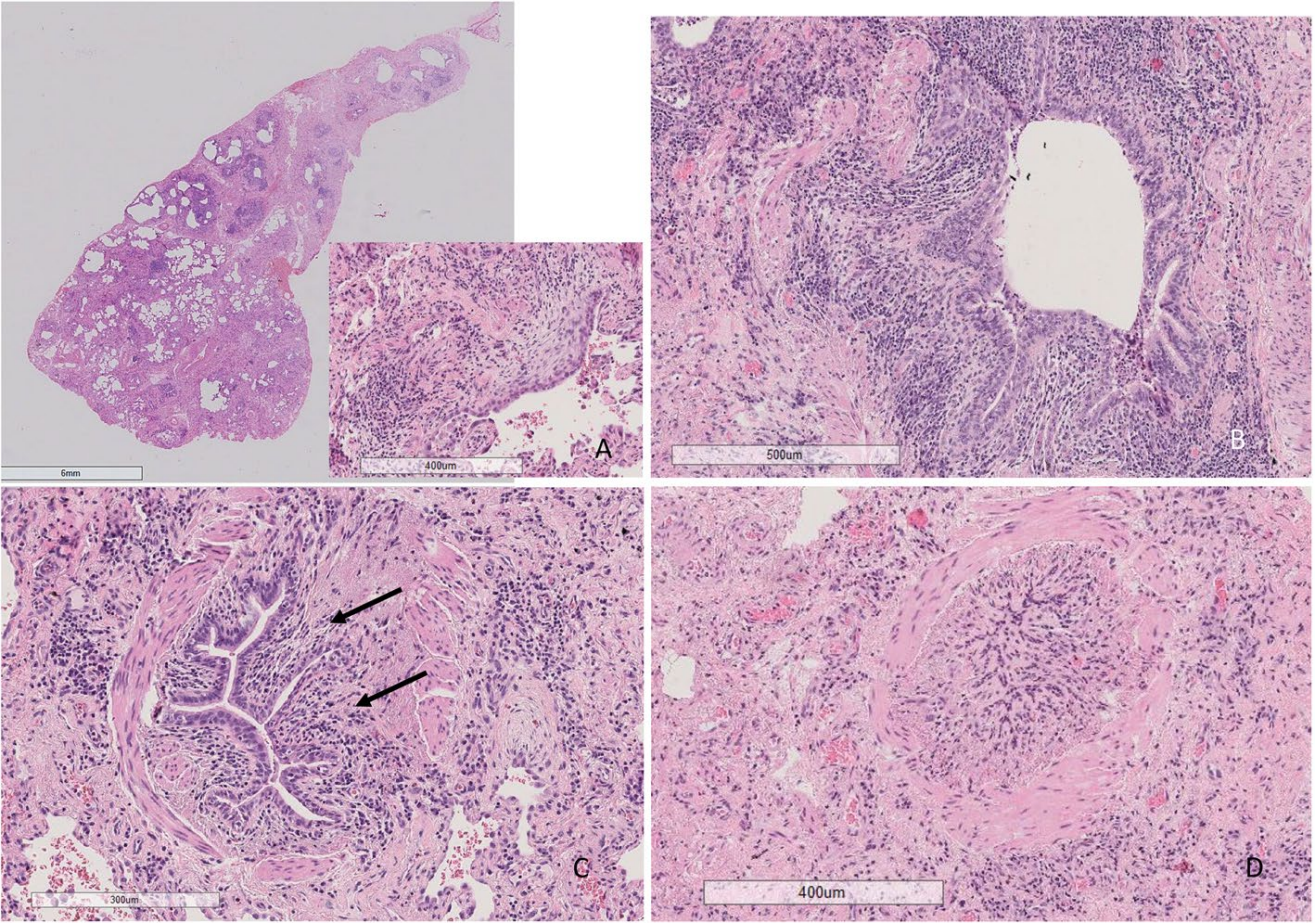
Histological images of the right lower lobe of the other patient with a *TERC* variant (no 2). There was wide architectural remodeling resembling UIP (**A**) with numerous fibroblast foci (insert). However, unlike in UIP, most of the bronchi and peribronchial tissue were heavily inflamed (**B**), many showed constricting fibrosis (arrows) (**C**), and some were obliterated (**D**). UIP = usual interstitial pneumonia

The patients (*n* = 16) who had undergone surgical lung biopsy (SLB) operation were younger than patients without SLB (59.3 and 66.7 years, respectively (*p* = 0.005)) while no difference in gender, smoking status, radiological patterns, clinical diagnoses or pulmonary function tests was found. Sixteen SLB samples and one autopsy sample, accounting for 25% of all patients, were re-evaluated according to the IPF 2022 guidelines [22]. Histopathological patterns were classified as indeterminate for UIP (8/47.1%), UIP (7/41.2%) or both probable UIP and alternative diagnosis in one patient each. Additional histological features were observed in 9 out of the 17 patients, including UIP combined with constrictive bronchiolitis and PPFE combined with granulomas (Table 4). The results of histological UIP pattern analysis and additional histological feature, radiologic UIP pattern, patient clinical diagnosis and genetic variant data are shown in Table 4 for the patients for whom histological investigations were available (Table 4). The histological and radiological images of the patients with pathological TERC variant are shown in Figs. 3, 4 and 5.

**Table 4:** Histopathologic, radiologic and clinical findings of the patients with familial pulmonary fibrosis with histologic investigation of lung tissue

Case	Genetic testing	Histological patterns and additional features	Histological Categorization 2022	Radiological Categorization 2022	Clinical diagnosis
1.	*TERC*	UIP, constrictive bronchiolitis	Indeterminate for UIP	Probable UIP	IPF
2.	^a^	UIP	UIP	UIP	IPF
3.	^a^	UIP	UIP	UIP	IPF
4.	*TERT*	UIP, HP, chronic inflammation	Indeterminate for UIP	Probable UIP	IPF
5.	^b^	UIP, HP, chronic inflammation	Indeterminate for UIP	Indeterminate for UIP	IPF
6.	^a^	UIP (mild), RB-ILD	UIP	Indeterminate for UIP	IPF
7.	^a^	UIP, bronchiolocentric fibrosis	Indeterminate for UIP	Probable UIP	IPF
8.	^a^	UIP, bronchiolocentric fibrosis	Indeterminate for UIP	UIP	IPF
9.	^b^	UIP, chronic inflammation, bronchiolocentric fibrosis	Indeterminate for UIP	Probable UIP	CTD-ILD
10.	^a^	Unclassified fibrosis	Alternative	Probable UIP	IPF
11.	^a^	UIP	UIP	UIP	IPF
12.	^a^	UIP, HP	Indeterminate for UIP	UIP	IPF
13.	*TERC*	Granulomatous reaction/ subacute and chronic HP, PPFE	Indeterminate for UIP	UIP	Unclassified
14.	^a^	UIP	UIP	UIP	IPF
15.	*RTEL1*	UIP, organizing pneumonia	UIP	Indeterminate for UIP	IPF
16.	*TERT*	UIP	UIP	UIP	IPF
17.	^a^	UIP (focal changes)	Probable UIP	Alternative diagnosis	Fibrotic NSIP

Cases 1-16 surgical lung biopsies, case 17 autopsy

*CTD-ILD* connective tissue disease associated interstitial lung disease, *HP* hypersensitivity pneumonia, *IPF* idiopathic pulmonary fibrosis, *NSIP* nonspecific interstitial pneumonia, *PPFE* pleuroparenchymal fibroelastosis, *RBILD* respiratory bronchiolitis interstitial lung disease, *RTEL1* regulator of telomere elongation helicase 1, *TERC* telomerase RNA component, *TERT* telomerase reverse transcriptase, *UIP* usual interstitial pneumonia

^a^Genetic testing not done

^b^Genetic testing done resulted in no known pathogenic variants

### Survival and risk prediction models

Half (34/50%) of the participants died (*n* = 29) or were lung-transplantated (*n* = 5) by the end of the follow-up with a median survival of 39.9 (20.5‒59.3) months. The overall survival of the study cohort was 78.3 (71.0‒85.6) months. Baseline pulmonary function data were available for 66 patients for whom risk prediction analyses could be performed. GAP stages I, II and III included 46 (67.6%), 18 (26.5%) and 2 (2.9%) patients, respectively. Stages II and III were combined into one group because of the small size of stage III. The CPI and GAP stage predict survival significantly (Fig. 3). According to the univariate analysis , the baseline DLCO% had a statistically significant influence on survival, which remained in a multivariate analysis with an HR of 0.95 (95.0% CI 0.92–0.99), while age, smoking status, FVC% predicted, FEV1% predicted, BMI or pathogenic gene variant did not have a significant influence in either univariate or multivariate analyses.

**Fig. 6:**
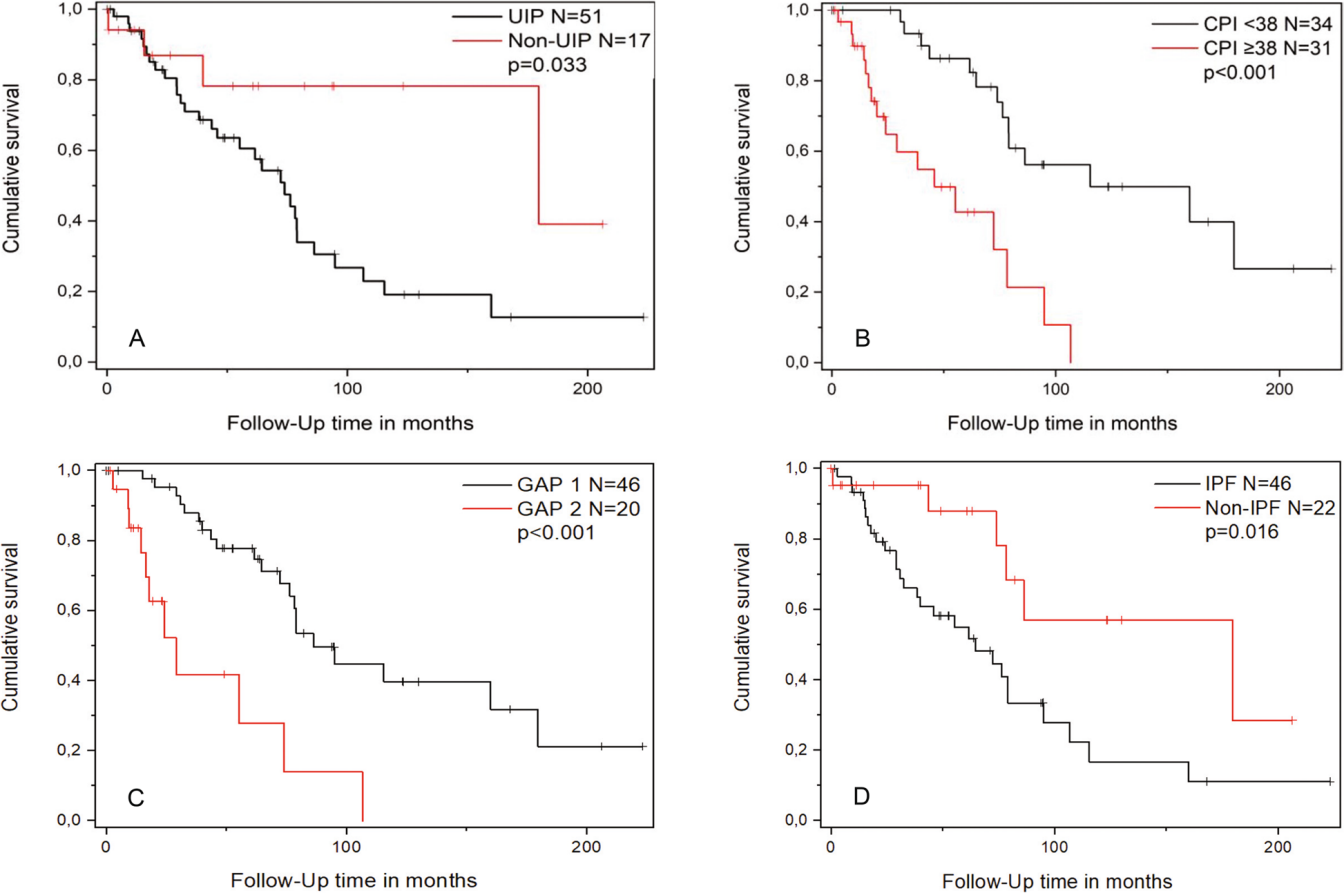
Kaplan-Meier survival curves (**A**) GAP 1 (GAP stage I) vs. GAP 2 (combined GAP stages II and III). **B** CPI groups as median value 38 was set as the cut-off. **C** UIP vs. non-UIP group. **D** IPF vs. non-IPF group. CPI = composite physiology index, GAP = gender-age-physiology -index, IPF = idiopathic pulmonary fibrosis, UIP = usual interstitial pneumonia

UIP group had worse survival (73.9 (55.9‒91.8) months) compared to non-UIP group (179.6 (0‒377) months, *p* = 0.033). Furthermore, IPF patients had worse survival compared to non-IPF group, as the overall median survival times were 64.5 (32.8‒96.2) months and 179.6 (40.9‒318.4) months, respectively (*p* = 0.016) (Fig. 6). Four IPF patients had deceased or been lung-transplanted before pirfenidone was permitted in Finland in 2013 and altogether six IPF patients before permission of nintedanib in 2015. When including IPF patients from 2013 onwards, those who were treated with antifibrotic medication (*n* = 27) did not have better survival compared to IPF patients without antifibrotics (*n* = 14).

## Discussion

In this retrospective study, we characterized a cohort of FPF patients in Northern Finland. In addition to clinical information, HRCT scans and histopathology were re-evaluated and classified according to the present international guidelines [22]. Furthermore, the functionality of the survival prediction models was also tested. We found that there was no male or smoking predominance in FPF patients, that both the radiological and histopathological findings were diverse, and that prediction models could be used for FPF patients. To the best of our knowledge, this study of 68 FPF patients is one of the largest published Northern European cohorts to date and included re-evaluated HRCT scans from all the patients.

Our results supported earlier studies showing that disease onset in FPF patients occurred earlier than that usually observed in IPF patients, at least in men [4, 19]. In the study of Krauss et al., the FPF patients were 58.3 years old, and in the study of Bennet et al., the men with familial IPF were 58.5 years old at the time of diagnosis [5, 19]. In our study, the men were significantly younger than the women (61.7 and 68.6 years, respectively; *p*<0.003), and the mean age was 64.4 years. Similarly, in the study of Froidure et al., the median age of the FPF population was 65 years [27]. In our study, age was not associated with survival, which may be explained by the fact that men had more often UIP pattern although it did not reach significant difference. Similarly to the results of Bennett et al. and Hodgson et al., there was no predominance of males in our study [19, 20]. According to both Krauss et al. and Steele et al., more than fifty percent of the patients were former or current smokers, and Kropski et al. suggested smoking as a risk factor for FPF [2, 5, 28]. In the study of Froidure et al., more than 75% of FPF patients had a smoking history, but they had fewer pack-years than the IPF group [27]. Cutting et al. reported that familial IPF patients had a shorter smoking history than sporadic IPF patients, which is in line with our study in which 54.4% of the patients were nonsmokers [4]. In our study, however, the nonsmokers were predominantly women (67.7% vs 43.2%, *p* = 0.043).

We were able to find only two previously published European studies on comorbidities in FPF patients [5, 19]. In both of the abovementioned studies, there were fewer patients than in our study (27 and 46 patients, respectively). Our results showed that hypercholesterolemia, CAD, hypertension and gastroesophageal reflux disease were present in at least 20% of the patients, similar to the findings of Bennett and coauthors. Interestingly, nineteen percent of the FPF patients in our study suffered from asthma, which was not observed in previous studies. In the recently published study on IPF, the most common pulmonary comorbidities were COPD (37%) and lung cancer (3%), while the most common nonpulmonary comorbidities were GERD, dyslipidemia and hypertension [15]. We found no COPD patients and only one patient with suspected lung cancer, which is a unique finding and may be due to the predominance of nonsmokers in our study, although the prevalence of nonpulmonary comorbidities in our study was similar to that in Lee’s study [15]. As in our findings, CAD and hypertension were the most common comorbidities in Finnish IPF patients regardless of their smoking status [29].

Earlier studies have shown that although IPF is the most common clinical phenotype in FPF, the radiological patterns are diverse. Lee et al. reported that UIP was the most common HRCT pattern in 22% of the cases [30]. while in the study of Bennet et al. UIP was found in 54% of the patients [19]. Cecchini et al studied 44 FPF patients, including 26 with shortened telomeres and 7 with telomere-related gene variants revealing that probable UIP was the most common pattern in the shortened telomere group, while indeterminate for UIP was the most common pattern in the group with telomeres >10^th^ percentile [10]. In the study of Diaz de Leon et al., 39 CT scans of patients with *TERT* gene variants with pulmonary fibrosis were evaluated; 74% had a typical UIP pattern, and the other patterns were consistent with UIP without honeycombing and atypical UIP [31]. By using the IPF guideline 2022, we also observed diverse radiological findings although the findings of our study were not straightforwardly comparable to those of other studies due to the different classification criteria. Interestingly, 13.2% of our patients exhibited PPFE in a single or combined pattern, which has not been shown in previous studies.

According to the study of Cecchini et al., IPF was the most common clinical diagnosis, although CHP was more common in patients with telomeres > the 10^th^ percentile [10]. Our clinical diagnoses were similar to those of Cutting et al., who reported IPF as the predominant clinical diagnosis, followed by unclassifiable PF [4]. Six patients in our study with shortened telomeres had UIP or combined UIP with PPFE patterns. Indeterminate for UIP was the most common pattern in histologic samples (8/47.1%), UIP (7/41.2%), and of probable UIP and alternative diagnoses, one of each was found.

Cecchini et al found that patients with shortened telomeres in particular had atypical histopathological findings [10]. On our histological re-evaluation, we found that unusual combinations of histological patterns existed in some patients. Interestingly, there were histological features of two patients with the *TERC* gene variant, one of which presented with a combination of UIP and constrictive bronchiolitis and the other with a combination of PPFE and granulomatous inflammation with fibrosis. Among 29 *TERT* gene variant patients, Diaz de Leon et al. reported a histologic UIP pattern in 89% of the patients but also additional histologic features, including chronic inflammation, scattered histiocytes and non-necrotizing granulomas [31]. Cecchini and coauthors analyzed the histological patterns of patients with shortened telomeres and revealed that although the UIP pattern was the most common, other patterns also existed, similar to the results of our study [10]. van Batenburg and coauthors compared the extent of inflammatory cells and fibrosis in the lung tissues of FPF and IPF patients and found no differences [32]. According to our reanalysis of histological features, however, in some cases, the extent of inflammation was high. In our experience, histological diversity may cause diagnostic problems and difficulties in classification, but it can also suggest FPF in an appropriate clinical context.

Cutting et al showed that FPF patients had shorter survival than patients with the sporadic form of the same clinical diagnosis, while familial IPF was associated with the shortest survival [4]. In our study, the median survival time of the deceased or lung-transplanted patients was 39.9 (20.5‒59.3) months, which was nearly the same as that of the IPF group in the studies of Newton et al. (3.75 years), in the *TERT* gene variant group of Diaz de Leon et al. (3 years, mean) and the *TERT* or *TERC* gene variant group of Borie et al. (4.2 years) [31, 33, 34]. The overall estimated survival, 78.3 (71.0‒85.6) months, was slightly worse than the survival reported by Bennett et al. for FPF patients (7.31 years) [19]. Planas-Cerezales et al. investigated the GAP index in patients with shortened telomeres and reported that patients younger than 60 years had the worst survival, although they were categorized into the GAP I stage [11]. In contrast, we found that the GAP I group had better survival than the combined GAP II and III groups. Furthermore, the CPI was significantly related to survival as a median value of 38 was set as the cut-off point.

Limitations of the study include its retrospective nature and small sample size; however, this study is one of the largest FPF studies in Northern Europe. Because the FPF does not have a specific ICD-10 code, the cohort might not be completely comprehensive, and some patients may be missing. Missing genetic testing information for most of the patients is a weakness of our study. Twenty-four patients underwent genetic testing, which was due to the clinical practice of the hospital and the retrospective nature of the study protocol; these patients included FPF patients from the year 2000 onwards. Systematic genetic testing of FPF patients started in our hospital in 2017; therefore, most of the patients were not tested due to the point of time of their illness. A few PFTs were missing, but HRCT was available from every patient. A quarter of the patients underwent histological investigation of lung tissue, which enabled us to perform reanalysis of histological features in a subset of the patients. The strengths of this study were the accurate collection of data and the radiological and histopathological re-evaluations provided by expert physicians.

## Conclusions

This study confirmed the results of earlier studies showing that FPF patients’ radiological and histopathological features are diverse and that histological features include unusual patterns and their combinations. All pathogenic gene variants found were related to the telomerase-associated genes.

## Data Availability

The data generated during the current study are available from the corresponding author upon reasonable request.

## Abbreviations

ASO: arteriosclerosis
ATS: American Thoracic Society
BMI: body mass index
CAD: coronary artery disease
CHP: chronic hypersensitivity pneumonitis
CI: cerebral infarction
COP: cryptogenic organizing pneumonia
COPD: chronic obstructive pulmonary disease
CPI: composite physiology index
CTD: connective tissue disease
CTD-ILD: connective tissue disease associated interstitial lung disease
DIP: desquamative interstitial pneumonia
DLCO: diffusion capacity to carbon monoxide
ERS: European Respiratory Society
FEV1: forced expiratory volume in one second
FPF: familial pulmonary fibrosis
FVC: forced vital capacity
GAP: gender-age-physiology
GERD: gastroesophageal reflux disease
HC: hypercholesterolemia
HF: heart failure
HP: hypersensitivity pneumonia
HRCT: high-resolution computer tomography
HT: arterial hypertension
ICD-10: International Classification of Diseases 10th edition
IIP: idiopathic interstitial pneumonia
ILD: interstitial lung disease IPF idiopathic pulmonary fibrosis
IPF: idiopathic pulmonary fibrosis
NSIP: nonspecific interstitial pneumonia
OP: organizing pneumonia
OSA: obstructive sleep apnea
*PARN*: poly(A)-specific ribonuclease
PF: pulmonary fibrosis
PFT: pulmonary function test
PPFE: pleuroparenchymal fibroelastosis
RA: rheumatoid arthritis
RB-ILD: respiratory bronchiolitis interstitial lung disease
*RTEL1*: regulator of telomere elongation helicase 1
SLB: surgical lung biopsy
*TERC*: telomerase RNA component
*TERT*: telomerase reverse transcriptase
UIP: usual interstitial pneumonia
